# Where to draw the line? The influence of prior relationship, perpetrator‐target sex and perpetrator motivation on the point at which behavior ‘crosses the line’ and becomes stalking

**DOI:** 10.1002/bsl.2592

**Published:** 2022-09-05

**Authors:** Adrian J. Scott, Sofia Stathi, Victoria Burniak

**Affiliations:** ^1^ Department of Psychology Goldsmiths, University of London London UK; ^2^ Institute for Lifecourse Development, School of Human Sciences University of Greenwich London UK

**Keywords:** perceptions, perpetrator motivation, perpetrator‐target sex, prior relationship, stalking

## Abstract

The present study examines the influence of prior relationship (intimate, non‐intimate), perpetrator‐target sex (male‐female, female‐male) and perpetrator motivation (romance, upset) on (1) the point at which behavior crosses the line and becomes stalking, and (2) the likelihood of offering five forms of advice to the target (formal support, informal support, protective measures, avoidance measures, threatening action). The study used a 2 × 2 × 2 between‐participants experimental design. Four‐hundred and sixty‐one UK students read one of eight versions of a hypothetical scenario that they were informed may or may not depict a stalking situation. Analyses revealed that 97.8% (*n* = 451) of participants believed the perpetrator's behavior constituted stalking, and that behavior was perceived to cross the line earlier in the scenario when the perpetrator's motivation was to upset the target in the context of a non‐intimate prior relationship only. Prior relationship, perpetrator‐target sex and perpetrator motivation also influenced the likelihood of offering various forms of advice to the target. These findings further demonstrate the impact of situational characteristics on perceptions of stalking and highlight the importance of educational campaigns and programs to increase people's understanding of stalking.

## 1 INTRODUCTION

Although there is no universal definition, the term stalking is generally used to refer to a pattern of behavior in which a person makes repeated attempts to impose unwanted communication and/or contact on another person (Mullen, et al., 1999, 2001). Common behaviors include unwanted calls, messages, emails, watching, following and approaching (Buhi et al., 2009; Melton, 2012; Rosay et al., 2010). National estimates suggest that a significant proportion of women and men experience stalking victimization. For example, the Crime Survey for England and Wales estimated that 19.9% of women and 9.6% of men had been stalked during their lifetime, and that 4.6% of women and 2.5% of men had been stalked during the previous 12 months (Office for National Statistics, 2020).

These estimates are concerning given the protracted nature of stalking and the harms associated with stalking victimization. When asked about their most recent experience of stalking, 53.3% of women and 50.8% of men reported that it had lasted for more than 6 months (Australian Bureau of Statistics, 2017). Furthermore, victims of stalking have been found to experience a range of social and psychological harms (e.g., reclusivity, helplessness and reduced self‐esteem), and have made substantial lifestyle changes (e.g., changes to routine, security and employment) because of their victimization (Blaauw et al., 2002; Pathé & Mullen, 1997; Spitzberg, 2002). In more severe cases, victims of stalking have experienced powerlessness, post‐traumatic stress disorder symptoms, and considered or attempted to take their own lives (Korkodeilou, 2016; Logan & Walker, 2019; Pathé & Mullen, 1997).

Stalking affects a significant proportion of the public, but UK and US estimates suggest that fewer than half of victims report their victimization to the police (Baum et al., 2009; Walby & Allen, 2004). Reasons for the non‐reporting of crime include perceptions of the situation being trivial or private, and of the police being disinterested or unable to help (Baum et al., 2009; Office for National Statistics, 2017). The internalization of myths that present stalking as a type of courtship behavior and minimize the associated harms (De Fazio et al., 2015; Ervin, 2015; McKeon et al., 2015) may also reduce the likelihood of victims reporting their experiences to the police.

It is apparent that beliefs and perceptions are important in determining whether a particular pattern of behavior is considered reasonable pursuit behavior or unreasonable stalking behavior (Mullen et al., 1999), and a body of research has examined the influence of various personal and situational characteristics on perceptions of stalking. Most of this research has adopted a mock juror or vignette methodology whereby participants are presented with a stalking scenario (a scenario that may or may not depict a stalking situation) that manipulates the situational characteristics of interest. Participants then respond to a series of items relating to their verdict decision, and/or their perceptions of the situation, the perpetrator and the target. Situational characteristics of direct relevance to the present study include prior relationship between the perpetrator and the target (prior relationship), sex of the perpetrator and the target (perpetrator‐target sex), and the underlying reason for the perpetrator's behavior (perpetrator motivation).

### 1.1 Literature review

#### 1.1.1 Prior relationship

Research has demonstrated that situations are often perceived to be more serious and to have a greater impact on the target when the perpetrator and target are strangers or acquaintances rather than ex‐partners. Hills and Taplin (1998) examined perceptions from a target perspective (where a particular pattern of behavior was directed toward the participant) with an Australian community sample, and found that participants believed they would experience more fear and would be more likely to call the police when the perpetrator was a stranger rather than an ex‐partner. Further research examining perceptions from an observer perspective (where a particular pattern of behavior was directed toward another person) with Australian, Malaysian, UK and US student and community samples found that participants were more likely to believe that a situation constituted stalking (Cass, 2011; Chung & Sheridan, 2021; Phillips et al., 2004; Scott et al., 2015; Scott, Rajakaruna et al., 2014; Sheridan et al., 2003), required police intervention (Chung & Sheridan, 2021; Scott et al., 2015; Scott, Rajakaruna et al., 2014; Sheridan et al., 2003) and warranted a criminal conviction (Scott et al., 2015; Scott, Rajakaruna et al., 2014), when the perpetrator was a stranger or acquaintance rather than an ex‐partner. This research also found that participants were more likely to believe the target would experience alarm and fear of violence when the perpetrator was a stranger or acquaintance (Chung & Sheridan, 2021; Scott et al., 2015; Scott, Rajakaruna et al., 2014). Research with UK police samples produced similar findings. Police officers were more likely to believe that a situation constituted stalking or harassment (Scott et al., 2013; Sheridan et al., 2016; Weller et al., 2013), required police intervention (Scott et al., 2013; Sheridan et al., 2016) and would last longer (Weller et al., 2013) when the perpetrator was a stranger rather than an acquaintance or ex‐partner. Police officers were also more likely to believe the target would experience alarm and fear of violence when the perpetrator was a stranger (Scott et al., 2013; Sheridan et al., 2016).

It is important to acknowledge that some studies with Australian and US student and community samples have not found an association between prior relationship and perceptions of stalking (Dennison & Thomson, 2000; Kinkade et al., 2005). Furthermore, another study with an Australian community sample has found that participants are more likely to believe a situation constitutes stalking when it is possible that the perpetrator's behavior is coincidental (occurring on the target's way to work, in a café and in a movie theater), and the perpetrator and target are ex‐partners rather than strangers (Dennison & Thomson, 2002). Scott, Rajakaruna et al. (2014) argued that these inconsistent findings likely reflect methodological differences, including the presence of ceiling effects (98% of the sample identified the pattern of behavior as stalking; Dennison & Thomson, 2000), and additional contextual information (the perpetrator in the ex‐partner condition was described as being possessive in the relationship; Dennison & Thomson, 2002), as well as the absence of a ‘true’ stranger condition (the perpetrator and target were described as meeting at a high school reunion or as having dated previously; Kinkade et al., 2005).

The finding that situations are often perceived to be more serious and to have a greater impact on the target when the perpetrator and target are strangers is concerning when compared to the reality of stalking victimization. Research using victim surveys (Galeazzi et al., 2009; Podaná & Imríšková, 2016), police reports (Belfrage & Strand, 2009; Rosay et al., 2010) and court reports (Malsch, 2007) has shown that most victims of stalking knew the perpetrator prior to being stalked. Research has also shown that ex‐partners are more likely to threaten and to use violence than strangers or acquaintances (e.g., Churcher & Nesca, 2013; James & Farnham, 2003; McEwan, 2017; Rosenfeld, 2004; Rosenfeld & Lewis, 2005; White et al., 2022).

Authors often refer to the just‐world hypothesis when interpreting the discrepancy between perceptions and reality, whereby people are motivated to view the world as a safe place and to attribute responsibility to the victims of crime to maintain this belief (Lerner, 1997; Lerner & Simmons, 1966). In the context of stalking, several studies have found that participants are more likely to believe the target is responsible for the situation when the perpetrator is an ex‐partner rather than a stranger (e.g., Chung & Sheridan, 2021; Scott et al., 2010; Scott & Sheridan, 2011; Sheridan et al., 2003). Thus, the target is perceived to share responsibility for what is considered a less serious situation between ex‐partners in a domestic setting that the perpetrator and target should resolve themselves (Sheridan et al., 2003). Importantly, misperceptions of this type may reduce the likelihood of victims, support networks and law officials identifying experiences of ex‐partner stalking, and reduce the likelihood of perpetrators being charged and prosecuted (see Scott, 2020).

#### 1.1.2 Perpetrator‐target sex

Research with Australian, Canadian, UK and US student and community samples has found no association between perpetrator‐target sex and perceptions of whether behavior constitutes stalking (Cass, 2011; Finnegan et al., 2018; Kinkade et al., 2005; Phillips et al., 2004; Sheridan et al., 2003). However, it has demonstrated that situations are more likely to be considered serious and to have a greater impact on the target when the perpetrator is a man and the target is a woman rather than vice versa. It is important, therefore, to distinguish perceptions of whether a particular situation constitutes stalking from perceptions of whether a situation is serious and will impact on the target.

Sheridan et al. (2003) examined perceptions from an observer perspective with a UK student sample and found that participants were more likely to believe that a situation required police intervention and would result in the target being injured, and were less likely to believe the target could help alleviate the situation, when the perpetrator was a man and the target was a woman. Further research with Canadian and US student samples found that participants were more likely to be concerned about the safety of the target (Finnegan & Timmons Fritz, 2012; Phillips et al., 2004), and to believe the target should seek informal (e.g., family and friends) and formal support (e.g., police) when the behavior was perpetrated by a man toward a woman (Cass & Mallicoat, 2015; Finnegan & Timmons Fritz, 2012; Phillips et al., 2004). Research with a Canadian police sample produced similar findings (Finnegan et al., 2018). Police officers were more likely to believe that a situation would cause the target emotional, psychological, physical and financial harm when the perpetrator was a man and the target was a woman.

The finding that situations are perceived to be more serious and to have a greater impact on the target when the perpetrator is a man and the target is a woman is again concerning when compared to the reality of stalking victimization. Although research using victim surveys (Galeazzi et al., 2009; Purcell et al., 2002), police reports (Lyon, 2006; Rosay et al., 2010) and court reports (Malsch, 2007) consistently demonstrates that stalking is predominantly perpetrated by men toward women, there is evidence to suggest that the behavior and its impact is similar irrespective of the sex of the perpetrator and target (e.g., Carabellese et al., 2013; Purcell et al., 2001; Sheridan et al., 2014; Strand & McEwan, 2011, 2012).

Authors often refer to gender‐role stereotypes when interpreting the discrepancy between perceptions and reality, whereby men are positioned as dominant and threatening, and women are positioned as weak and vulnerable (Gerber, 1991). Thus, a particular pattern of behavior is more likely to be considered serious and to have an impact on the victim when the perpetrator is a man and the victim is a woman rather than vice versa (e.g., Cass & Mallicoat, 2015; Cass & Rosay, 2012; Finnegan & Timmons Fritz, 2012), because men are perceived to be more threatening than women and women are perceived to be more vulnerable than men. Consistent with prior relationship, misperceptions of this type may reduce the likelihood of victims, support networks and law officials identifying experiences of stalking, and of perpetrators being charged and prosecuted, in situations where the perpetrator is a woman and the victim is a man (see Scott, 2020).

#### 1.1.3 Perpetrator motivation

No research to date has examined the influence of perpetrator motivation on perceptions of stalking. However, a small body of closely related research with Australian community samples has examined the influence of perpetrator intent (Dennison, 2007; Dennison & Thomson, 2000, 2002). In this context, perpetrator intent relates to explicit evidence of intent to create fear or apprehension or to cause mental or physical harm, and was manipulated via the presence or absence of a threatening message (Dennison, 2007; Dennison & Thomson, 2000, 2002) and the discovery of the alleged perpetrator's diary detailing the target's movements (Dennison & Thomson, 2000, 2002). This research found that participants were more likely to believe that a situation constituted stalking (Dennison & Thomson, 2002), constituted illegal behavior, and would be repeated (Dennison, 2007) when there was explicit evidence of intent. Participants were also more likely to believe the target should call or threaten to call the police and press or threaten to press charges (Dennison, 2007) when there was explicit evidence of intent. Although Dennison and Thomson (2000) found no association between perpetrator intent and perceptions of whether the behavior constitutes stalking, this likely reflected the presence of ceiling effects as outlined previously.

The finding that situations are more likely to be perceived to constitute stalking and illegal behavior when there is explicit evidence of intent have important implications for the differentiation of reasonable pursuit behavior from unreasonable stalking behavior. For example, overreliance on perpetrator intent may reduce the likelihood of identifying experiences of stalking, and of perpetrators being charged and prosecuted, in the absence of explicit evidence of intent. Building on these, we examine whether perpetrator motivation influences the point at which behavior crosses the line and becomes stalking, and the likelihood of offering specific advice to the target. This will help shed further light on the effects of situational characteristics on possible stalking situations.

## 2 PRESENT STUDY

Previous research has shown that prior relationship, perpetrator‐target sex and perpetrator intent influence perceptions of stalking. However, this research has focused on the influence of these characteristics in the context of relational stalking, where the perpetrator is always pursuing a relationship with the target. In the present study, we provide a more nuanced examination of prior relationship and perpetrator‐target sex by exploring their influence in relational contexts (where the perpetrator's behavior is motivated by the desire to romance the target) and non‐relational contexts (where the perpetrator's behavior is motivated by the desire to upset the target). Furthermore, previous research has examined the influence of personal and situational characteristics on perceptions of stalking using standard mock juror and vignette methodologies. Although appropriate, these standard methodologies have only been used to examine *if* a particular pattern of behavior constitutes stalking.

The present study extends prior research by using a modified vignette methodology to examine, for the first time, whether prior relationship, perpetrator‐target sex and perpetrator motivation influence (1) the point at which behavior crosses the line and becomes stalking, and (2) the likelihood of offering five forms of advice to the target (formal support, informal support, protective measures, avoidance measures, threatening action). We developed the modified vignette methodology, so that we could examine the influence of these situational characteristics on *if and when* a particular pattern of behavior shifts from ‘reasonable’ pursuit behavior to ‘unreasonable’ stalking behavior. It is important to examine these shifts in perception because the longer it takes a victim to identify their experiences as stalking, the more likely the perpetrator's behavior is to escalate and the less likely the victim is to develop and use effective coping strategies.

In contrast to previous research, where perceptions have been examined from a target or observer perspective, the present study examines perceptions of stalking from a friend perspective (where a particular pattern of behavior was directed toward an imaginary friend of the participant). Furthermore, the hypothetical scenario used in the present study was presented sequentially in the form of three separate conversations with this imaginary friend. A friend perspective was chosen because research has shown that victims of stalking often approach friends and family for support in the first instance (Buhi et al., 2009; Galeazzi et al., 2009; Purcell et al., 2002). Thus, the beliefs and perceptions of friends, and the advice they offer, may be influential in determining what victims of stalking do next (Scott, 2020). The sequential presentation of the hypothetical scenario was chosen because we wanted participants to indicate if, and if so when, they believed the perpetrator's behavior crossed the line and became stalking before they had read the scenario in its entirety. Finally, three separate conversations were chosen because we wanted the scenario to have an overarching narrative, whereby the imaginary friend discussed their situation with participants over a protracted period of time.

### 2.1 Method

#### 2.1.1 Participants and design

Participants were recruited between October 2017 and November 2018 via UK university participation schemes (68.7%, *n* = 317), online participant panels (25.4%, *n* = 117) and social media (5.9%, *n* = 27). Participants recruited via university participation schemes received course credit, participants recruited via online panels received a small monetary payment and participants recruited via social media did not receive any compensation. The initial sample comprised 580 participants, but in line with the focus of the study, non‐students (*n* = 65) and non‐UK residents (*n* = 54) were removed prior to analysis. The final sample comprised 461 students, resident in the UK: 361 (78.3%) women, 98 (21.3%) men, and 2 (0.4%) who identified as other, with an average age of 22.83 years (*SD* = 7.29, 18–62 years). Most participants were studying psychology or a closely related subject (75.1%, *n* = 346), with remaining participants studying a variety of subjects including business, computer science, engineering, mathematics and medicine (18.7%, *n* = 86), or preferring not to say (6.3%, *n* = 29). Ninety‐six (20.8%) participants reported having experienced stalking victimization and 19 (4.1%) participants reported having engaged in stalking perpetration. Although not a focus of the present study, a series of additional univariate analyses revealed that participant sex and previous stalking experience (victimization and/or perpetration) did not influence perceptions of the point at which behavior crosses the line and becomes stalking, or the likelihood of offering five forms of advice to the target.

Participants were randomly allocated to one of eight experimental conditions, reflecting the use of a 2 × 2 × 2 between‐participants design. The independent variables comprised prior relationship (intimate, non‐intimate), perpetrator‐target sex (male‐female, female‐male) and perpetrator motivation (romance, upset). The dependent variables comprised whether the perpetrator's behavior constituted stalking (yes, no), and if so, the point in the scenario where it crossed the line and became stalking (sentence number, 1–46, see Appendix A for examples); as well as the likelihood of advising the target to seek/take formal support, informal support, protective measures, avoidance measures and threatening action (factor analyzed scale items, 1 not at all likely to 10 extremely likely). Each experimental condition included 53 to 63 participants: 7–19 men, 38–52 women and 0–1 other.

#### 2.1.2 Materials

##### Scenario

Participants read one of eight versions of a hypothetical scenario (representing the different levels of prior relationship, perpetrator‐target sex and perpetrator motivation) that were presented sequentially in the form of three separate conversations with an imaginary friend (the target). The scenario incorporated a range of behaviors that are listed in the Protection of Freedoms Act (2012) as being associated with stalking, but may appear routine and/or harmless in isolation. Two examples of the hypothetical scenario are provided in Appendix A.

Participants were asked to imagine that the first conversation occurred a short while after the target started university and describes how an ex‐partner/fellow student (the perpetrator) has been watching the target and has approached them. They were then asked to imagine that the second conversation took place a few weeks after the first conversation and describes how the perpetrator obtained the target's number, how they have been receiving text messages and silent phone calls from a withheld number, and how the perpetrator left a note asking them to meet in person. Finally, participants were asked to imagine that the third conversation took place a month after the second conversation and describes how the target changed their number and how this temporarily improved the situation; how the perpetrator befriended one of the target's housemates, and how the perpetrator spent time at their house, and tried to stop them from leaving the kitchen.

Prior relationship was manipulated so that the perpetrator was an ex‐partner of the target (intimate) or a fellow student who attends some of the target's lectures (non‐intimate). Perpetrator‐target sex was manipulated so that the perpetrator was male (Michael) and the target was female (Emily) or vice versa. The perpetrator motivation conditions were developed after consideration of previous research examining perpetrator motives (Johnson & Thompson, 2016; McFarlane & Bocij, 2003), and involved the manipulation of the perpetrator's verbal and non‐verbal behavior so that it reflected their desire to romance the target (e.g., ‘…you're amazing…’, ‘You know you're the only one for me’ and the giving of longing looks) or to upset the target (e.g., ‘…you're so pathetic…’, ‘You know things won't end well for you’ and the giving of nasty looks).

##### Where to draw the line

Participants were asked to indicate whether they believed the situation constituted stalking at the end of each conversation (yes, no), unless they had already indicated yes at the end of a previous conversation. If participants responded yes, they were asked to indicate the point in the scenario where the perpetrator's behavior crossed the line and became stalking. Heat map questions with hidden regions were used so that participants could click anywhere in the conversation and the corresponding sentence number would be recorded for the purpose of analysis.

##### Advice to the target

Having read all three conversations, participants were asked to indicate how likely they would be to offer 16 forms of advice to the target. The advice items were developed after consideration of previous research examining how victims of stalking respond to their victimization (Arnocky & Vaillancourt, 2014; Geistman et al., 2013; Storey & Hart, 2011), and were measured using 11‐point scales, ranging from 0 (not at all likely) to 10 (extremely likely). Given the number of advice items, principal components analysis with varimax rotation was performed prior to analysis. Five factors were identified and labeled: formal support, informal support, protective measures, avoidance measures and threatening action. One item (‘Seek professional help from a counselor?’) was removed from the protective measures factor because it did not align with the other items in the factor (Clark‐Carter, 2019). The 16 advice items (organized by factor) are provided in Appendix B.

Averages were calculated for the items loading onto each factor for the purpose of analysis. Formal support comprised five items advising the target to avoid speaking with the perpetrator, to gather evidence, to apply for a restraining order, and to contact the police and/or a victimization support group (Cronbach's *α* = 0.73); informal support comprised two items advising the target to tell family and friends, and to ask them to ask the perpetrator to stop (*r* = 0.19, *p* < 0.001); protective measures comprised four items advising the target to stay at home, avoid certain areas, formulate a safety plan and take additional safety measures (Cronbach's *α* = 0.62); avoidance measures comprised two items advising the target to ignore the perpetrator or do nothing (*r* = 0.22, *p* < 0.001); and threatening action comprised two items advising the target to threaten the perpetrator or to ask family and friends to threaten the perpetrator to make them stop (*r* = 0.53, *p* < 0.001).

##### Demographic information

Finally, participants were asked to provide basic demographic information, including their sex (male, female, other), age (years), student status (yes, no), country of residence (UK, other), stalking victimization (yes, no, prefer not to say) and stalking perpetration (yes, no, prefer not to say). The student status and country of residence items were included so that we could exclude any non‐students and non‐UK residents in line with the focus of the study.

#### 2.1.3 Procedure

The study was hosted via Qualtics XM (www.qualtrics.com), an online survey development platform. Participants were presented with an information letter that informed them that they would read a hypothetical scenario that may or may not depict a stalking situation. They then provided consent before being randomly presented with one of eight versions of the scenario using the block randomizer function. Each hypothetical scenario was presented sequentially in the form of three separate conversations with the target. Participants were asked to read each conversation in turn, and to indicate whether they believed that the situation constituted stalking. If they responded yes at the end of a particular conversation, they were asked to indicate the point in the scenario where the perpetrator's behavior crossed the line and became stalking, and to read the remaining conversations. Having read all three conversations, participants were asked to complete the advice items, by indicating the likelihood of offering 16 forms of advice to the target. Finally, participants were asked to provide some basic demographic information before being thanked and presented with a debrief statement. The study received approval from the university ethics committee and was conducted in accordance with the ethical requirements of the British Psychological Society.

### 2.2 Results

All data were entered, checked and analyzed using IBM SPSS Statistics Version 27. Parametric analyses are reported despite several normal distribution and homogeneity of variance assumption violations because the analyses are considered robust given the size of the sample (Tabachnick & Fidell, 2013) and given that separate non‐parametric analyses revealed a consistent pattern of univariate significance.

#### 2.2.1 Where to draw the line

Each scenario comprised three conversations and 46 sentences: the first conversation presented sentences 1–15, the second conversation presented sentences 16–29 and the third conversation presented sentences 30–46 (see Appendix A for examples). Descriptive analyses revealed that 97.8% (*n* = 451) of participants believed that the perpetrator's behavior constituted stalking, and 2.2% (*n* = 10) did not. Of participants who believed the perpetrator's behavior constituted stalking, 40.1% (*n* = 181) believed it crossed the line during the first conversation, 52.5% (*n* = 237) believed it crossed the line during the second conversation, and 7.3% (*n* = 33) believed it crossed the line during the third conversation. Three separate chi‐square analyses were performed to examine whether prior relationship, perpetrator‐target sex and perpetrator motivation influenced whether participants believed the perpetrator's behavior constituted stalking during the first, second or third conversations; and none were statistically significant.

**FIGURE 1:**
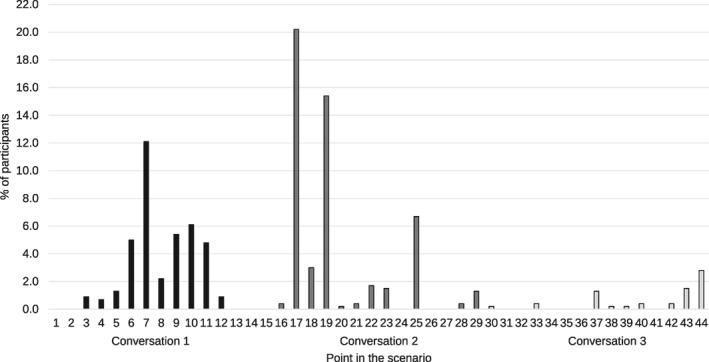
The point in the scenario (the sentence number) where participants believed the behavior crossed the line and became stalking

Further descriptive analyses examined the point in the scenario where participants believed that the perpetrator's behavior crossed the line and became stalking, and revealed a mean sentence number of 16.47 (*SD* = 9.24), a median and mode of 17 and a range of 3–44. Figure 1 displays the point in the scenario where participants believed that the perpetrator's behavior crossed the line and became stalking. During the first conversation, participants were most likely to believe the perpetrator's behavior crossed the line when the context changed from the target seeing the perpetrator during lectures to seeing them during lunch (sentence 7), and when the perpetrator sat behind the target during a seminar (sentence 9) and proceeded to tap them on the shoulder (sentence 10). During the second conversation, participants were most likely to believe the perpetrator's behavior crossed the line when the perpetrator got hold of the target's number (sentence 17), when the perpetrator presumably sent text messages and made silent phone calls to the target (sentence 19) and when the perpetrator left a note in the target's locker asking them to meet in person (sentence 25). During the third conversation, participants were most likely to believe the perpetrator's behavior crossed the line when the perpetrator secretly befriended one of the target's housemates (sentence 37), and when the perpetrator waited for the target in their kitchen (sentence 43) and proceeded to try and stop the target from leaving the kitchen (sentence 44).

A 2 × 2 × 2 ANOVA was performed to examine the influence of prior relationship, perpetrator‐target sex and perpetrator motivation on the point at which the behavior crossed the line and became stalking. The analysis revealed a significant interaction effect for prior relationship and perpetrator motivation, *F*(1, 443) = 7.57, *p* = 0.006, *η* ^2^ = 0.02. Separate *t*‐test analyses revealed that the perpetrator's behavior was perceived to cross the line earlier in the scenario when their motivation was to upset the target (*M* = 14.62, *SD* = 9.01) rather than to romance the target (*M* = 17.94, *SD* = 8.79) in the context of a non‐intimate relationship, *t*(223) = −2.79, *p* = 0.006, Cohen's *d* = 0.37. However, perpetrator motivation did not significantly influence perceptions in the context of an intimate relationship (*M* = 17.23, *SD* = 9.34 vs. *M* = 15.83, *SD* = 9.59), *t*(224) = 1.11, *p* = 0.27, Cohen's *d* = 0.15. No other main or interaction effects were significant.

### 2.3 Advice to the target

Descriptive analyses revealed that participants were most likely to advise the target to seek informal support (*M* = 6.80, *SD* = 2.03) and formal support (*M* = 6.58, *SD* = 2.03). They were less likely to advise the target to take protective action (*M* = 4.70, *SD* = 1.97), and were least likely to advise the target to take avoidance measures (*M* = 2.91, *SD* = 2.07) or threatening action (*M* = 1.86, *SD* = 2.11).

A 2 × 2 × 2 MANOVA was performed to examine the influence of prior relationship, perpetrator‐target sex and perpetrator motivation on the five advice factors. The analysis revealed a significant interaction effect for perpetrator‐target sex and perpetrator motivation, *F*(5, 449) = 2.34, *p* = 0.041, *η* ^2^ = 0.03, as well as significant main effects for prior relationship, *F*(5, 449) = 4.01, *p* = 0.001, *η* ^2^ = 0.04, perpetrator‐target sex, *F*(5, 449) = 9.29, *p* < 0.001, *η* ^2^ = 0.09 and perpetrator motivation, *F*(5, 449) = 5.81, *p* < 0.001, *η* ^2^ = 0.06. Additional ANOVAs using Bonferroni corrected alpha values of 0.01 were therefore performed. The *F* ratios, significance values, means and standard deviations are displayed in Tables 1 and 2.

**TABLE 1:** Multivariate and univariate analyses of variance F ratios for advice to the target by prior relationship, perpetrator‐target sex and perpetrator motivation

		ANOVA				
	MANOVA	Formal support	Informal support	Protective measures	Avoidance measures	Threatening action
Variable	*F*	*F*	*F*	*F*	*F*	*F*
Prior relationship (PR)	4.01**	3.28	2.91	2.55	10.27**	2.52
Perpetrator‐target sex (P‐TS)	9.29***	25.58***	1.48	28.69***	1.10	9.03**
Perpetrator motivation (PM)	5.81***	15.64***	8.98**	0.10	0.84	4.01
PR × P‐TS	0.99	2.63	0.08	0.28	0.46	0.15
PR × PM	1.84	0.96	5.11	0.00	1.20	0.23
P‐TS × PM	2.34*	0.93	1.44	5.14	3.21	3.66
PR × P‐TS × PM	0.69	0.16	0.17	0.77	2.06	0.00

*Note*: *F* ratios are Wilk's Lambda approximations of *F*s.

Abbreviations: ANOVA, univariate analysis of variance; MANOVA, multivariate analysis of variance.

Bonferroni corrected alpha value = 0.01.

*p < 0.05, **p < 0.01, ***p < 0.001.

**TABLE 2:** Means and standard deviations for advice to the target as a function of prior relationship, perpetrator‐target sex and perpetrator motivation

	Advice to the target									
	Formal support		Informal support		Protective measures		Avoidance measures		Threatening action	
Condition	*M*	*SD*	*M*	*SD*	*M*	*SD*	*M*	*SD*	*M*	SD
Prior relationship										
Intimate	6.44	2.11	6.94	2.06	4.55	1.94	3.21_a_	2.15	2.01	2.23
Non‐intimate	6.73	1.93	6.65	2.00	4.84	1.99	2.62_a_	1.94	1.71	1.98
Perpetrator‐target sex										
Male‐female	7.03_a_	1.82	6.91	1.96	5.16_a_	1.80	2.82	1.97	2.15_a_	2.19
Female‐male	6.11_a_	2.12	6.68	2.11	4.21_a_	2.02	3.02	2.16	1.56_a_	1.98
Perpetrator motivation										
Romance	6.24_a_	2.06	7.07_a_	2.02	4.74	2.09	2.99	2.02	2.05	2.13
Upset	6.94_a_	1.93	6.51_a_	2.02	4.65	1.83	2.83	2.11	1.66	2.08

*Note*: For prior relationship, perpetrator‐target sex and perpetrator motivation, column means sharing subscripts are significantly different (*p* < 0.01).

Although there was a significant interaction effect in the MANOVA, there were no significant interaction effects in the subsequent ANOVAs after Bonferroni correction. Regarding significant main effects, participants were more likely to advise the target to take avoidance measures when the prior relationship was intimate (*M* = 3.21, *SD* = 2.15) rather than non‐intimate (*M* = 2.62, *SD* = 1.94), *F*(1, 453) = 10.27, *p* = 0.001, *η* ^2^ = 0.02. Participants were also more likely to advise the target to seek formal support (*M* = 7.03, *SD* = 1.82), to take protective measures (*M* = 5.16, *SD* = 1.80) and to take threatening action (*M* = 2.15, *SD* = 2.19) when the perpetrator was a man and the target was a woman rather than vice versa (*M* = 6.11, *SD* = 2.12, *M* = 4.21, *SD* = 2.02 and *M* = 1.56, *SD* = 1.98, respectively), *F*(1, 453) = 25.58, *p* < 0.001, *η* ^2^ = 0.05, *F*(1, 453) = 28.67, *p* < 0.001, *η* ^2^ = 0.06, and *F*(1, 453) = 9.03, *p* = 0.003, *η* ^2^ = 0.02, respectively. Finally, participants were more likely to advise the target to seek formal support (*M* = 6.94, *SD* = 1.93), and were less likely to advise the target to seek informal support (*M* = 6.51, *SD* = 2.02), when the perpetrator's motivation was to upset the target rather than to romance the target (*M* = 6.24, *SD* = 2.06 and *M* = 7.07, *SD* = 2.02, respectively), *F*(1, 453) = 15.64, *p* < 0.001, *η* ^2^ = 0.03 and *F*(1, 453) = 8.98, *p* = 0.003, *η* ^2^ = 0.02, respectively.

## 3 DISCUSSION

Stalking is difficult to define because it incorporates a range of unwanted behaviors over a protracted period of time, and perceptions are integral to determining whether a particular pattern of behavior is identified as stalking (Mullen et al., 1999, 2001; Scott, 2020; Spitzberg & Cupach, 2007). We provided participants with a hypothetical scenario that was presented sequentially in the form of three separate conversations, and asked them to indicate *if* and *when* the perpetrator's behavior crossed the line and became stalking. Our findings showed that nearly all participants believed the perpetrator's behavior constituted stalking, and that the perpetrator's behavior crossed the line when the context changed (e.g., from seeing the perpetrator during lectures to seeing them during lunch, from seeing the perpetrator in a university setting to seeing them in their home setting); when the perpetrator contacted the target (e.g., when the perpetrator sent text messages and made silent phone calls, when the perpetrator left a note in the target's locker); and when the perpetrator approached the target (e.g., when the perpetrator sat behind the target during a seminar, when the perpetrator waited for the target in their kitchen). These findings may reflect differences in the perceived legitimacy of the perpetrator's behavior across different contexts. For example, the perpetrator may have a legitimate reason for attending lectures, being at university and for looking at the target. However, they do not have a legitimate reason for attending the same lunch venue, for getting hold of the target's number and contacting them via phone, or for befriending one of the target's housemates and approaching them at home. Regarding advice to the target, participants were more likely to advise formal, informal and protective measures than they were to advise avoidance measures or threatening action. These findings are reassuring as previous research suggests that proactive methods, including informal and formal support, represent effective coping strategies (Chan & Sheridan, 2020a, 2020b).

### 3.1 Prior relationship

Prior relationship was found to have minimal impact on perceptions of stalking in the present study, with just one significant difference relating to the likelihood of advice to the target: participants were more likely to recommend avoidance measures in the context of an intimate rather than a non‐intimate prior relationship. Although the minimal impact of prior relationship is inconsistent with much of the previous research (Cass, 2011; Phillips et al., 2004; Scott et al., 2015; Scott, Rajakaruna et al., 2014; Sheridan et al., 2003), it is consistent with the findings of Kinkade et al. (2005) and may reflect the use of an educational setting. The perpetrator in the non‐intimate condition was described as a fellow student (a peer) of the target and may not have been perceived as a ‘true stranger’. This distinction is important in the context of the just‐world hypothesis because the target may have been perceived to share responsibility for what is considered to be a less serious situation between acquaintances in an educational setting. Sheridan et al. (2003) argued that behavior is more likely to be considered reasonable when perpetrated by an acquaintance rather than a stranger because of the false belief that the victim triggered the behavior. Consistent with this argument, research with a UK student sample found that participants were more likely to attribute partial responsibility to the target because of something they did or failed to do when the perpetrator was an acquaintance rather than a stranger (Scott, Gavin, 2014). Importantly, the attribution of blame and responsibility to victims of stalking reduces the likelihood of them receiving appropriate advice and support, and increases the risk of escalation.

### 3.2 Perpetrator‐target sex

Perpetrator‐target sex influenced perceptions of the likelihood of advice to the target. However, it did not influence perceptions of when behavior crossed the line and became stalking. Regarding advice to the target, participants were more likely to recommend formal support, protective measures and threatening measures when the perpetrator was a man and the target was a woman. It is concerning that participants were less likely to advise the target seek formal support or take protective measures when the behavior was perpetrated by a woman toward a man, given evidence to suggest that behavior and its impact is similar irrespective of the sex of the perpetrator and target (e.g., Carabellese et al., 2013; Purcell et al., 2001; Sheridan et al., 2014; Strand & McEwan, 2011, 2012). When related to previous research, these findings align with the common misperception that situations are not serious when behavior is perpetrated by a woman toward a man (Finnegan et al., 2018; Finnegan & Timmons Fritz, 2012; Phillips et al., 2004; Sheridan et al., 2003) and may be understood with reference to gender‐role stereotypes (Gerber, 1991). From this perspective, the seeking of formal support and taking protective measures may be considered an overreaction when a ‘dominant and threatening’ man is targeted by a ‘weak and vulnerable’ woman.

Although non‐significant, the findings for where to draw the line, align with previous research that suggests there is no difference in perceptions of whether behavior constitutes stalking (Cass, 2011; Finnegan et al., 2018; Kinkade et al., 2005; Phillips et al., 2004; Sheridan et al., 2003). In combination, research suggests people are equally capable (or incapable) of identifying potential stalking situations irrespective of perpetrator‐target sex, but that they underplay the impact of these situations when the target is a man rather than a woman. These misperceptions may prevent men from taking their victimization seriously, and from seeking support and taking protective measures. However, research with a Hong Kong student sample suggests few significant sex differences (7 differences across 40 comparisons) in the coping strategies of self‐reported victims of stalking (Chan & Sheridan, 2020a). Overall, men and women were similarly likely to use the most frequently adopted coping strategies of ignoring the problem, involving others to resolve the problem, and behaving cautiously; whereas men were more likely than women to use some of the least frequently adopted coping strategies of negotiating with the perpetrator, and threatening or using physical violence against the perpetrator.

### 3.3 Perpetrator motivation

Perpetrator motivation influenced perceptions of when behavior crossed the line and became stalking, and the likelihood of advice to the target. Regarding perceptions of where to draw the line, participants believed that behavior crossed the line and became stalking earlier in the scenario when the perpetrator's behavior was motivated by the desire to upset the target in the context of a non‐intimate prior relationship only. Consistent with the just‐world hypothesis, the non‐significant finding for perpetrator motivation in the context of an intimate prior relationship may reflect people's tendency to blame the target and mitigate the behavior of the perpetrator when they are ex‐partners because of their shared history (Scott, Gavin et al., 2014; Scott, Rajakaruna et al., 2014). It may also reflect participants' tendency to romanticize the perpetrator's behavior when the perpetrator and target were ex‐partners, irrespective of whether the perpetrator's behavior was manipulated to reflect the desire to romance or upset the target. By romanticizing the perpetrator's behavior in this way, participants widen the range of pursuit behaviors considered reasonable and increase sympathy toward the perpetrator (Gavin & Scott, 2016). Importantly, the normalization of stalking situations reduces the likelihood of victims, support networks and law officials from identifying experiences of stalking, and again, increases the risk of escalation.

Regarding advice to the target, participants were more likely to recommend formal advice, but were less likely to recommend informal advice, when the perpetrator's behavior was motivated by the desire to upset the target. These findings suggest that perpetrator motivation impacts upon the perceived severity of the situation, and aligns with previous research that has shown that a perpetrator's behavior is more likely to be considered serious when they intend to cause the target fear or harm (Dennison, 2007; Dennison & Thomson, 2002; Phillips et al., 2004).

### 3.4 Implications, limitations and further research

The present study employed a modified vignette methodology to provide a more nuanced examination of situational characteristics by exploring the influence of prior relationship, perpetrator‐victim sex and perpetrator motivation on the point at which behavior crosses the line and becomes stalking, and the likelihood of offering five forms of advice to the target. The term stalking is often used lightly, with little consideration of its seriousness or of the associated harms (Boehnlein et al., 2020), and perceptions are “…integral to determining whether victims identify their own experiences as stalking, and whether support networks and law officials identify other people's experiences as stalking” (Scott, 2020, p. 246). Therefore, misperceptions may reduce the likelihood of early intervention, and increase the risk of escalation (Scott, 2020; White et al., 2022).

There is general agreement that education is required to address common misperceptions regarding stalking (Brewster, 2001; Cheyne & Guggisberg, 2018; Korkodeilou, 2016; Ogilvie, 2000; Spence‐Diehl & Potocky‐Tripodi, 2001), and it is important that findings from this and other perception research are incorporated into general awareness campaigns (e.g., UK National Stalking Awareness Week), school education programs (e.g., UK Relationships and Sex Education), and the training of frontline police officers to reduce the influence of stereotypes and myths, and increase people's understanding of stalking (see Page & Scott, 2021). Consideration of *how* and *why* personal and situational characteristics influence *if* and *when* behavior is perceived to cross the line and become stalking will also help victims, support networks and law officials identify experiences of stalking and ultimately help support, respond to and protect victims of stalking.

It is important to acknowledge that the present study was limited by use of a heterosexual scenario, and a predominantly female student sample from a Western, Educated, Industrialized, Rich and Democratic (WEIRD) country. Therefore, it would be worthwhile to examine the influence of perpetrator‐target sex in the context of same and opposite sex scenarios to establish whether stereotypes regarding sexual orientation drive beliefs and perceptions of stalking. Participant sex was not a focus in the present study, and a student sample was chosen because stalking is particularly prevalent within this population (Brady et al., 2017). However, further appropriately powered research is necessary to determine the generalizability of the non‐significant univariate analyses for participant sex, and to examine whether participant sex interacts with other situational characteristics to influence perceptions of the point at which behavior crosses the line and becomes stalking, or the likelihood of offering advice to the target. Further research is also necessary to determine whether the findings of this and other perception research generalize to non‐student, non‐WEIRD country samples. Chan and Sheridan (2017) highlighted the need for research to examine cultural influences on perceptions of stalking, and Chung and Sheridan (2021) found that Malaysian participants were less likely than English participants to believe that behavior constituted stalking or required police intervention.

It is also important to acknowledge that the present study was limited by the framing and construction of the scenario. Participants were informed that they would read a hypothetical scenario that may or may not depict a stalking situation, before being asked to indicate whether they believe the situation constitutes stalking. Although it was not possible to avoid framing the study in terms of stalking given its focus, it is likely that use of the term stalking heightened participants' awareness and influenced subsequent responses. Future research could address this limitation by removing all references to stalking and simply asking participants how they would respond to their hypothetical friend in this situation. Furthermore, although the scenario incorporated a range of behaviors that are listed in the Protection of Freedoms Act (2012) as being associated with stalking, it was not developed to legally constitute stalking. Therefore, it was not possible to determine the accuracy of participants' perceptions regarding the point at which the perpetrator's behavior crossed the line and became stalking in legal terms. Further research using scenarios depicting legally defined stalking situations via the modified vignette methodology developed for the present study is necessary to examine the influence of various personal and situational characteristics on the accuracy of participants' perceptions.

Finally, the construction of the scenarios meant that perpetrator motivation was manipulated via the perpetrator's verbal and non‐verbal behavior, and that participants read each conversation in its entirety before indicating if and when the perpetrator's behavior crossed the line and became stalking. Consequently, some participants would have perceived the perpetrator's behavior differently (e.g., motivated by a desire for control or revenge), and some participants would have been influenced by information presented after the point where they decided to draw the line. Future research could address these limitations by explicitly stating the perpetrator's motivation and presenting scenarios one sentence at a time, rather than one conversation at a time.

## 4 CONCLUSION

The present study used a modified vignette methodology, for the first time, to examine the influence of prior relationship, perpetrator‐target sex and perpetrator motivation on the point at which behavior crosses the line and becomes stalking and the likelihood of offering five forms of advice to the target. The perpetrator's behavior was perceived to cross the line and become stalking earlier when the perpetrator was motivated by the desire to upset the target in the context of a non‐intimate prior relationship only. Furthermore, prior relationship, perpetrator‐target sex and perpetrator motivation influenced the perceived likelihood of offering various forms of advice to the target. It is important, therefore, that educational campaigns and programs incorporate the findings of this and other perception research to increase people's understanding of stalking.

## APPENDIX A

### A.1 Example scenario for the ‘female‐male, intimate, upset’ condition

#### A.1.1 Conversation 1

Imagine you're catching up with your good friend, Michael, as you have not seen him since he started university. You ask how things are going and he tells you the following: Yeah, things have been good.^1^ At least for the most part.^2^ I mean I'm really enjoying the university lifestyle, you know the subject is really interesting and I've met some great people.^3^ There is Emily though (an ex‐girlfriend of Michael), who attends some of my lectures.^4^ I'm not going to lie, she kind of makes me feel uncomfortable.^5^ Sometimes in lectures I feel like I'm being watched and when I turn around she seems to be looking at me.^6^ It's not just in lectures either, like when I go for lunch with friends I sometimes see her hanging around.^7^ I swear when I see her she gives me these nasty looks.^8^ The other day she sat right behind me during one of my seminars.^9^ I pretended not to notice, but she tapped me on the shoulder.^10^ When I turned around she said ‘I have no idea why I went out with you, you're so pathetic’.^11^ I honestly didn't know how to respond so I just asked her to be quiet.^12^ I'm seriously confused!^13^ Perhaps I should try to avoid her although I don't think it will be easy given that we're at the same uni.^14^ I guess I'll just remain calm and not let her get to me.^15^

#### A.1.2 Conversation 2

Now imagine you check up on Michael a few weeks later and ask how things are going. He tells you the following: Remember how last time I saw you I was telling you about Emily?^16^ Well, she's really starting to annoy me now, as she managed to get hold of my number.^17^ Apparently, she asked this guy I've been working on a project with and he gave it to her!^18^ Anyway, not long after I started receiving these silent phone calls from a withheld number.^19^ It's so irritating.^20^ I'm convinced it's her, I mean I sometimes receive them during lectures and whenever I get one she's just sitting there staring at her phone.^21^ To make things worse, I started receiving text messages from an unknown number that must be hers.^22^ The messages typically say stuff like: ‘Your life must suck because nobody likes you’.^23^ Oh and there's more.^24^ The other day when I went to get my stuff from my locker I found a note from her that read: ‘Meet me outside or you're going to regret it’.^25^ Stupid I know, but I decided to meet her.^26^ I thought if we talked I could make her understand that I just want to be left alone.^27^ Anyway when we spoke I asked her to stop bothering me.^28^ I don't think it helped though, as she became aggressive and started calling me names.^29^

#### A.1.3 Conversation 3

Now imagine Michael calls you a month later to update you on the ‘Emily’ situation. He tells you the following: So I've changed my number.^30^ Sorry I didn't let you know sooner, but things have been really hectic.^31^ Remember those silent phone calls and text messages I was getting?^32^ Well they got more frequent, to the point where they totally did my head in.^33^ Anyway, they stopped after I changed my number and things actually seemed to be okay for a while.^34^ I still saw Emily on campus now and then, but nothing happened so it started to feel like she'd finally got the message.^35^ But I was wrong.^36^ It turns out that during this time she'd been busy befriending one of my house mates, Kaitlin.^37^ You know, the girl who was always annoying me at the start of semester?^38^ Anyway, initially I thought I must be overreacting as Emily came to the house a couple of times and it was fine.^39^ She didn't try to talk to me or anything.^40^ Her and Kaitlin would meet at ours and then go out pretty soon after.^41^ However, this has now changed and she's spending more and more time at our house.^42^ A few days ago I got home to find her waiting for me in the kitchen.^43^ She tried to stop me from leaving and said ‘You know things won't end well for you’.^44^ I ended up staying at my friend's flat that night but have no idea what I should do.^45^ It's not as if I can stay at his flat every time she's at our house.^46^

### A.2 Example scenario for the ‘male‐female, non‐intimate, romance’ condition

#### A.2.1 Conversation 1

Imagine you're catching up with your good friend, Emily, as you have not seen her since she started university. You ask how things are going and she tells you the following: Yeah, things have been good.^1^ At least for the most part.^2^ I mean I'm really enjoying the university lifestyle, you know the subject is really interesting and I've met some great people.^3^ There is this one guy though, Michael, who attends some of my lectures.^4^ I'm not going to lie, he kind of makes me feel uncomfortable.^5^ Sometimes in lectures I feel like I'm being watched and when I turn around he seems to be looking at me.^6^ It's not just in lectures either, like when I go for lunch with friends I sometimes see him hanging around.^7^ I swear that when I see him he gives me these longing looks.^8^ The other day he sat right behind me during one of my seminars.^9^ I pretended not to notice, but he tapped me on the shoulder.^10^ When I turned around he said ‘I think you're amazing, we should go out sometime’.^11^ I honestly didn't know how to respond so I just asked him to be quiet.^12^ I'm seriously confused!^13^ Perhaps I should try to avoid him although I don't think it will be easy given that we're at the same uni.^14^ I guess I'll just remain calm and not let him get to me.^15^

#### A.2.2 Conversation 2

Now imagine you check up on Emily a few weeks later and ask how things are going. She tells you the following: Remember how last time I saw you I was telling you about that Michael guy?^16^ Well, he's really starting to annoy me now, as he managed to get hold of my number.^17^ Apparently, he asked this girl I've been working on a project with and she gave it to him!^18^ Anyway, not long after I started receiving these silent phone calls from a withheld number.^19^ It's so irritating.^20^ I'm convinced it's him, I mean I sometimes receive them during lectures and whenever I get one he's just sitting there staring at his phone.^21^ To make things worse, I started receiving text messages from an unknown number that must be his.^22^ The messages typically say stuff like ‘Please give me a chance and go out with me’.^23^ Oh and there's more.^24^ The other day when I went to get my stuff from my locker I found a note from him that read ‘Meet me outside or I'll keep asking you out’.^25^ Stupid I know, but I decided to meet him.^26^ I thought if we talked I could make him understand that I just want to be left alone.^27^ Anyway, when we spoke I asked him to stop bothering me.^28^ I don't think it helped though, as he became desperate and started begging me to reconsider.^29^

#### A.2.3 Conversation 3

Now imagine Emily calls you a month later to update you on the ‘Michael’ situation. She tells you the following: So I've changed my number.^30^ Sorry I didn't let you know sooner, but things have been really hectic.^31^ Remember those silent phone calls and text messages I was getting?^32^ Well they got more frequent, to the point where they totally did my head in.^33^ Anyway, they stopped after I changed my number and things actually seemed to be okay for a while.^34^ I still saw Michael on campus now and then, but nothing happened so it started to feel like he'd finally got the message.^35^ But I was wrong.^36^ It turns out that during this time he'd been busy befriending one of my house mates, Aaron.^37^ You know, the guy who was always annoying me at the start of semester?^38^ Anyway, initially I thought I must be overreacting as Michael came to the house a couple of times and it was fine.^39^ He didn't try to talk to me or anything.^40^ Him and Aaron would meet at ours and then go out pretty soon after.^41^ However, this has now changed and he's spending more and more time at our house.^42^ A few days ago I got home to find him waiting for me in the kitchen.^43^ He tried to stop me from leaving and said ‘You know you're the only one for me’.^44^ I ended up staying at my friend's flat that night but have no idea what I should do.^45^ It's not as if I can stay at her flat every time he's at our house.^46^

Note. Participants were not presented with the superscript numbers. They are presented here to illustrate the hidden regions used to record the corresponding sentence number.

## APPENDIX B

### B.1 Advice items organized by factors

#### B.1.1 Formal support

1. Contact the police?
2. Apply for a restraining/intervention order?
3. Contact a stalking victimization support group or agency?
4. Start documenting and gathering evidence of [the perpetrator's] behavior?
5. Speak with [the perpetrator] again and ask [them] to stop? (Item reverse scored to represent ‘Avoid speaking with [the perpetrator] again and asking [them] to stop?’)

#### B.1.2 Informal support

6 Tell family and other friends about the situation?
7 Ask family and friends to ask [the perpetrator] to stop?

#### B.1.3 Protective measures

8 Avoid certain areas, change routine?
9 Formulate a safety plan (a plan to help avoid dangerous situations)?
10 Stay at home?
11 Take additional security measures (e.g., self‐defense classes)?

#### B.1.4 Avoidance measures

12 Ignore [the perpetrator]?
13 Do nothing?

#### B.1.5 Threatening action

14 Threaten [the perpetrator] to make [them] stop?
15 Ask family and friends to threaten [the perpetrator] to make [them] stop?

Note. One item ('Seek professional help from a counselor?’) was removed from the protective measures factor because it did not align with the other items in the factor.
